# A diagnostic platform for rapid, simultaneous quantification of procalcitonin and C-reactive protein in human serum

**DOI:** 10.1016/j.ebiom.2022.103867

**Published:** 2022-02-08

**Authors:** Xiangkun Elvis Cao, Serge Y. Ongagna-Yhombi, Ruisheng Wang, Yue Ren, Balaji Srinivasan, Joshua A. Hayden, Zhen Zhao, David Erickson, Saurabh Mehta

**Affiliations:** a Sibley School of Mechanical and Aerospace Engineering, Cornell University, Ithaca, NY, United States; b Division of Nutritional Sciences, Cornell University, Ithaca, NY, United States; c Meinig School of Biomedical Engineering, Cornell University, Ithaca, NY, United States; d Department of Pathology and Laboratory Medicine, Weill Cornell Medicine, New York, NY, United States; e Institute for Nutritional Sciences, Global Health, and Technology, Cornell University, Ithaca, NY, United States

**Keywords:** Sepsis, Point-of-care diagnostics, Bacterial and viral infections, Multiplexed lateral flow assay, Procalcitonin (PCT), C-reactive protein (CRP)

## Abstract

**Background:**

Early and accurate determination of bacterial infections as a potential cause for a patient's systemic inflammatory response is required for timely administration of appropriate treatment and antibiotic stewardship. Procalcitonin (PCT) and C-reactive protein (CRP) have both been used as biomarkers to infer bacterial infections, particularly in the context of sepsis. There is an urgent need to develop a platform for simultaneous quantification of PCT and CRP, to enable the potential use of these biomarkers at the point-of-care.

**Methods:**

A multiplexed lateral flow assay (LFA) and a fluorescence optical reader were developed. Assay performance was validated by testing spiked antigens in the buffer, followed by a validation study comparing results with conventional assays (Roche Cobas e411 Elecsys PCT and Siemens ADVIA XPT CRP) in 25 archived remnant human serum samples.

**Findings:**

A linear regression correlation of 0·97 (*P* < 0·01) was observed for PCT, and a correlation of 0·95 (*P* < 0·01) was observed for CRP using direct patient samples. We also validated our platform's ability to accurately quantify high-dose CRP in the hook effect range where excess unlabeled analytes occupy binding sites at test lines.

**Interpretation:**

A fluorescence reader-based duplex LFA for simultaneous quantification of PCT and CRP was developed and successfully validated with clinical samples. The rapid, portable, and low-cost nature of the platform offers potential for differentiation of bacterial and viral infections in emergency and low-resource settings at the point-of-care.

**Funding:**

NIH/NIBIB Award 1R01EB021331, and Academic Venture Fund from the Atkinson Center for a Sustainable Future at Cornell University.

**Research in context**

**Evidence before this study**

Sepsis is a life-threatening syndrome arising from the host's extreme and dysregulated inflammatory response primarily to bacterial infections. Early and accurate differential diagnosis of bacterial and viral infections is required to timely administer appropriate antibiotic therapy and prevent antibiotic misuse. Current clinical diagnostics focus on detecting specific pathogen-induced host biomarkers to infer the presence of bacterial infections. Procalcitonin (PCT) and C- reactive protein (CRP) have both been widely used as biomarkers implying bacterial infections due to their predictable kinetics, appropriate half-life, and high specificity. The simultaneous quantification of PCT and CRP can enable the early detection of bacterial infections. However, most current central laboratory methods for quantifying PCT and CRP are highly dependent on expensive equipment and trained personnel, limiting their wide use in point-of-care settings. While there are studies and commercial strips for PCT or CRP detection, limited options are available to simultaneously detect and quantify PCT and CRP over a dynamic range observed in sepsis patients, especially for high-dose CRP in the hook effect range. Currently, there are no FDA-cleared point-of-care PCT assays available.

**Added value of this study**

The platform for simultaneous quantification of PCT and CRP reported in the current study has several competitive advantages (e.g., portability, rapid response, low cost), making it suitable for use in point-of-care clinical settings. Our clinical validation with patient samples covers the whole dynamic range observed in sepsis patients, and our quantification successfully navigates the hook effect for CRP. An added advantage is our custom-built UV fluorescence-based optical reader, which could pick up faint signals to significantly increase the limit of detection and quantification accuracy of our system.

**Implications of all available evidence**

Our method comparison using patient samples demonstrated good agreement with FDA- cleared methods for co-quantifying PCT and CRP. The rapid, portable, and low-cost nature of the platform offers great potential for bacterial and viral infections differentiation in low-resource settings at the point of care.

Alt-text: Unlabelled box

## Introduction

Sepsis is a life-threatening syndrome arising from the host's extreme and dysregulated inflammatory response primarily to bacterial infections. With around 50 million cases per year, sepsis leads to more than 11 million deaths and presents a significant health challenge associated with high mortality and morbidity worldwide.^1^ Each year, more than 1·7 million adults have sepsis in the United States, resulting in at least 270,000 deaths.^2^ The early and accurate diagnosis of sepsis is critical for prompt therapeutic intervention and is strongly emphasized in the “Surviving Sepsis Campaign” guidelines.^3^, ^4^, ^5^ A delayed diagnosis of sepsis can lead to prolonged and empirical administration of broad-spectrum antibiotics and promote bacterial selection and ultimately antibiotic resistance. Antibiotic resistance has severe implications for healthcare costs, public health, and most importantly, threatens the usefulness and continued reliance of antibiotics as primary therapeutic agents.^6^^,^^7^ During the COVID-19 pandemic, though most hospitalized patients do not have bacterial co-infections,^8^ elevated levels of biomarkers specific to bacterial infections (i.e., > 0·5 ng/mL for procalcitonin) are frequently observed for severe cases for patients with COVID infections,^9^^,^^10^ and procalcitonin measurement on admission has shown great value in identifying the risks for patients for bacterial co-infection at an early stage, enhancing the clinical management of the coronavirus.^11^

Blood culture has been widely accepted as the gold standard method for identifying the source of illness and confirmation of sepsis.^12^ However, this method has several drawbacks, such as the long time taken (typically > 24 h) for positive confirmation, and the possibility of false negatives.^13^ Molecular-based diagnostic assays provide promising options to rapidly probe for pathogen molecular signatures and host antibodies against pathogens.^14^ For example, the real-time multiplex polymerase chain reaction (PCR) test has been adopted as a quantitative approach to concurrently amplify and detect target DNA molecules within hours.^15^^,^^16^ Despite their success, most of these methods remain tedious, labour-intensive, and highly dependent on expensive equipment and trained personnel, limiting their wide use in point-of-care settings.^17^ Recent development in ultrafast photonic PCR has shown the potential to reduce the thermocycling time for traditional PCR systems.^18^, ^19^, ^20^ Paper-based microfluidic devices, such as the lateral flow assay (LFA), can further reduce the diagnostic cost while providing an actionable test result.^21^, ^22^, ^23^ To infer the presence of bacterial infections, current LFA test strips focus on detecting specific pathogen-induced host biomarkers. Among these biomarkers, procalcitonin (PCT) and C-reactive protein (CRP) have received the most attention.^24^^,^^25^ The increase in PCT and CRP levels during infection exhibits predictable kinetics, appropriate half-life, and high specificity, making them an ideal combination of biomarkers for the diagnosis of sepsis due to bacterial infections.

PCT is a 116 amino acid polypeptide synthesized by C-cells of the thyroid gland.^26^ In healthy individuals, the serum concentration of PCT is usually at a low concentration below 0·5 ng/mL, and it can exceed 10 ng/mL under septic shock.^27^ PCT undergoes several proteolytic cleavages that release three mature proteins, including calcitonin. Experimental sepsis models have demonstrated that PCT level systematically increases several orders of magnitude within 4 h in response to bacterial infection, peaking at 6 h with an 8–24 h plateau.^28^ Conversely, the PCT level drops when the infection subsides. The rapid and predictable response of PCT level to bacterial infections makes it a promising biomarker for diagnosing bacterial infections, assessing the severity of the disease, and monitoring the response to treatment.^29^

CRP is a pentameric protein of 224 amino acids secreted in the bloodstream by the liver cells in response to infection and inflammatory insults.^30^ Likewise, the baseline level of CRP is low in the sera of healthy individuals (< 1 mg/dL), but CRP increases several folds and can even reach > 25 mg/dL in response to severe bacterial infection and inflammation insults,^31^^,^^32^ with an onset of 12–24 h, and a 20–72 h plateau.^28^ Several studies have shown that CRP is a good biomarker for diagnosing bacterial infection and sepsis. The invaluable properties of PCT and CRP have led to their use in diagnostics as biomarkers of systemic inflammation and sepsis.^33^

PCT and CRP have both been used as biomarkers to differentiate bacterial and viral infections in clinical settings^34^^,^^35^ Compared to PCT, CRP is a more extensively studied biomarker but is less specific to bacterial infections.^36^ Compared to CRP, PCT has an earlier response to bacterial infections (i.e., the onset of within 4 h vs. 12–24 h) and a greater correlation with illness severity, but the physiological range is usually much lower than CRP (i.e., ng/mL vs. mg/dL). Therefore, PCT detection, especially during mild infections (e.g., local infection when PCT concentration is between 0·05 and 0·5 ng/mL), could pose a diagnostic challenge due to its low-circulating level. Thus, a diagnostic strategy that uses two biomarkers in synergy holds excellent potential for diagnosing bacterial infection, following the progress and outcome of treatment, and reducing antibiotic misuse.^36^^,^^37^ While the concept of combining PCT and CRP biomarkers has been demonstrated in three previous studies,^38^, ^39^, ^40^ one study was only able to show semi-quantitative detection by setting up positive/ negative cut-offs for PCT and CRP,^39^ and none was able to conduct clinical validation to prove the quantification accuracy of the relatively large dynamic range observed in sepsis patients: from 0·05 ng/mL to > 10 ng/mL for PCT, and from 1 mg/dL to > 25 mg/dL for CRP (which runs into the hook effect range). Moreover, the background disparity across multiple strip images can be evidently observed from the images directly taken by a fluorescent image reader,^40^ which further compromised the quantification accuracy. This also proved the need for a novel reader design to enable background consistency (which reduces intra-assay background disparity) and to pick up faint signals (which increases detection limit for target analytes).

In this study, we describe the development of an optical reader-based duplex LFA diagnostic platform for rapid and simultaneous quantification of PCT and CRP antigens over a large physiological range. The platform comprises a custom-built portable UV fluorescence optical reader based on a new imaging algorithm to reduce background disparity and to accurately quantify the fluorescence intensity of the LFA with ultra-sensitive europium chelate microspheres as the conjugation nanoparticles. The assay was optimised and assessed for robustness with purified recombinant PCT and CRP proteins spiked in the buffer, followed by method comparison using archived remnant human serum samples. Our fluorescence reader-based duplex LFA platform, as demonstrated to be in good agreement with FDA cleared methods, presents a rapid, accurate, and low-cost approach to quantify the two biomarkers inferring bacterial infections concurrently.

## Methods

### Ethics

We obtained de-identified clinical remnant samples, and this use was approved by the Weill Cornell Medicine Institutional Review Board for assay validation.

### Reagents and materials

Mouse anti-human PCT monoclonal antibodies (Detection: Cat # MBS310731, Clone # 42; Capture: Cat # MBS310737, Clone # 16B5) were purchased from MyBioSource, Inc. (San Diego, CA, USA). Mouse monoclonal anti-human CRP antibodies (Detection: Cat # M353, Lot # MA1293; Capture: Cat # M354, Lot # MA1459) were acquired from CalBioreagents (San Mateo, CA, USA). Anti-mouse IgG (whole molecule) antibody produced in goat (Cat # M8642-1 mg) was used as the control antibody and was obtained from Sigma-Aldrich, Inc. (St. Louis, MO, USA). The europium nanoparticle (EuNP) conjugation kit (Cat # 1200-0003) was acquired from Expedeon Ltd. (San Diego, CA, USA). Human procalcitonin recombinant protein (Cat # RP-75698) was purchased from Thermo Fisher Scientific (Waltham, MA, USA). C-reactive proteins (antigens) (Cat # 30-AC05, Cat # 30-AC05AF) were obtained from Fitzgerald Industries International (Acton, MA, USA). Purified recombinant human calcitonin peptide (Cat # 30-AC44) was acquired from Fitzgerald Industries International (Acton, MA, USA).

Distilled water (Water-Ultra-Pure^TM^, Cat # 10977–105, 500 mL) was purchased from Invitrogen/Life Technologies. Tris-buffered saline (TBS) buffer (Cat # 28358, 1 × 25 mM Tris, 0·15 M NaCl) was purchased from Thermo Fisher Scientific. Phosphate Buffered Saline (PBS) solution (Cat # BP399-500, 10X) was acquired from Fisher Bioreagents. 1 M HEPES solution (Cat # H0887, pH 7·0–7·6, 100 mL), Triton^TM^ X-100 BioXtra (Cat # T9284, 100 mL), Phosphate Buffered Saline with 10% Bovine Albumin (Cat # SRE0036, 250 mL), Sucrose BioXtra (Cat # S7903-250G, ≥ 99·5% GC), Bovine Serum Albumin (Cat # 7030-50 g, pH 7, purify ≥98%), and Tween®20 (Cat # P9416, 100 mL) was obtained from Sigma-Aldrich, Inc.

Syringes (Luer-Lock^TM^ tip (Cat # 30-9628) and Precision Glide^TM^ syringe needles (Cat # 305165) were sourced from BD (Franklin Lakes, NJ, USA). The cellulose fibre (Cat # CFSP203000), conjugate pad sheet (Cat # GFCP203000), Hi-Flow Plus 180 nitrocellulose membrane cards (Cat # HF180MC100), G041 glass fibre conjugate pad sheet (Cat # GFCP203000), and cellulose-based absorbent pad (Cat # CFSP20300) were purchased from EMD Millipore (Billerica, MA, USA).

### Equipment

An Automated Lateral Flow Reagent Dispenser (Cat # 07·882·00, *L* = 457 mm, *W* = 146 mm, *H* = 75 mm) by ClaremontBio Solutions (Upland, CA, USA) was used for printing test line and control line antibodies on the membrane. A syringe pump was mounted to the dispenser. An adjustable AC/DC Adapter converted 120 VAC to 3–12 VDC, and provided power for the syringe pump. A paper trimmer from Dahle North America, Inc. (Peterborough, NH, USA) was used to cut the nitrocellulose membranes into 4 mm × 6 cm strips.

### Antibody conjugation to europium nanoparticles

We conjugated detection antibodies to EuNPs using the conjugation kit according to the manufacturer's instructions. Briefly, stock antibodies were first diluted in 50 mM HEPES buffer (pH 7·0–7·6) at 1 mg/mL, followed by a 10-fold dilution in reaction buffer to 0·1 mg/mL. Antibody conditioned in dilution buffer (45 µL) was then transferred into a tube containing freeze-dried EuNPs, followed by 15 min incubation at room temperature. The conjugation reaction was terminated by adding 5 µL of 1X Quencher solution. The solution was left to quench for 5 min at room temperature before being transferred to a microcentrifuge tube. After 8 min of centrifugation at 13,800 g and subsequent removal of the supernatant, 40 µL of the resuspension buffer was added to the pellet to get a conjugate solution at the concentration of 1%. The conjugated antibody was immediately stored at 4 °C or diluted to a working concentration.

### Test strip architecture and testing protocol

**Figure 1:**
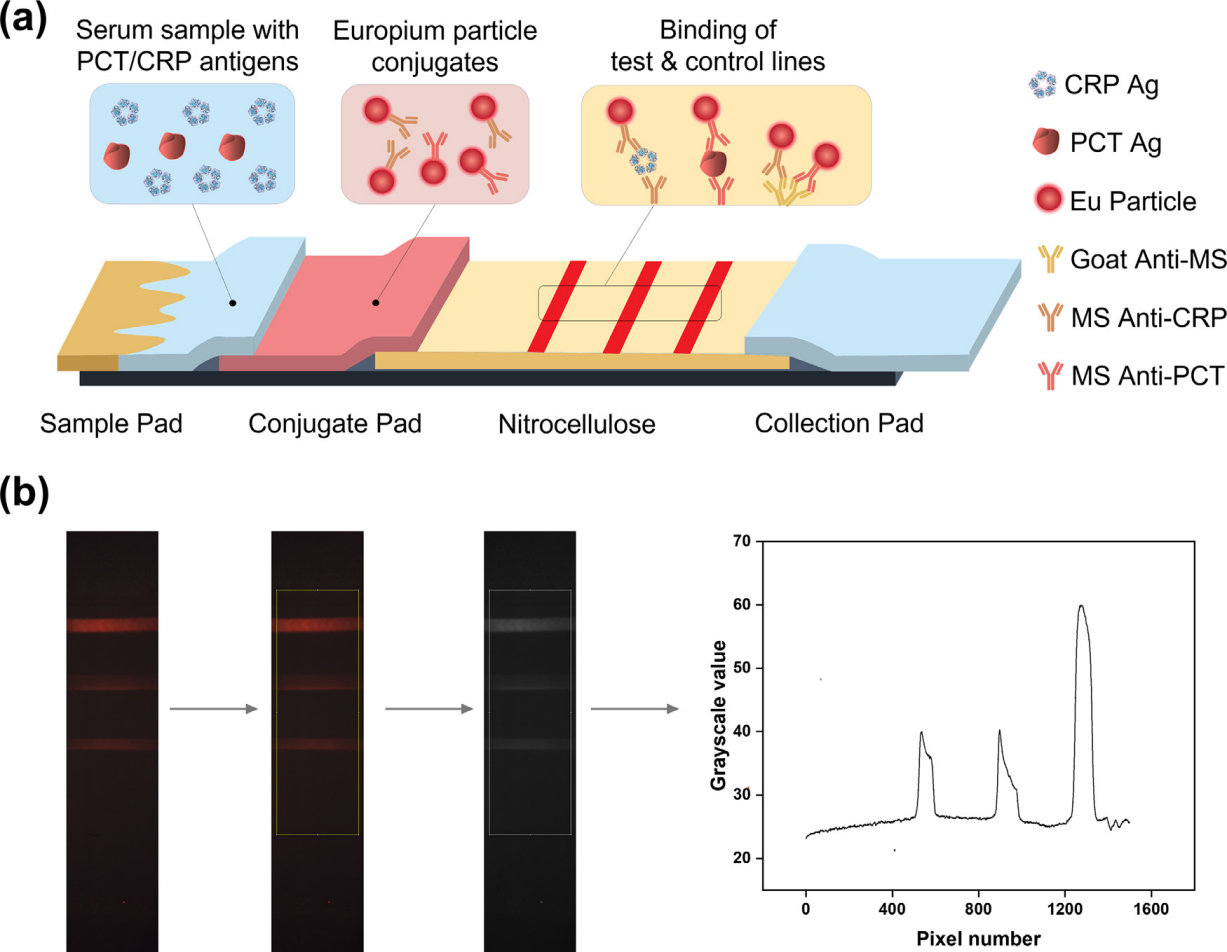
Schematics of the PCT/CRP duplex lateral flow assay (LFA) and signal intensity quantification protocol. (a) Schematics of the PCT/CRP duplex LFA. A serum sample containing PCT and CRP antigens is added to the sample pad (Sizes of PCT and CRP antigens not to scale). The conjugate pad contains both PCT and CRP detection antibodies pre-conjugated with EuNPs. PCT and CRP antigens will bind to EuNP-conjugated PCT and CRP detection antibodies, respectively. As the sample flows through the nitrocellulose pad from left to right, the europium-conjugated CRP detection antibodies and PCT detection antibodies are captured by the first line (CRP line) and the second line (PCT line), respectively. Unbound conjugates are captured by the third line (control line). (b) Image processing protocol to quantify signal intensities of test and control lines. The three lines shown in the figure represent the CRP test line, the PCT test line, and the control line, from bottom to top. The bottom of the region of interest represents pixel position at 0, and its top represents pixel position at 1499. The width of the region of interest is 500.

The LFA test strip consists of a sample pad, a conjugate pad, a nitrocellulose membrane, and a collection pad mounted on a plastic backing with overlap between each component, as shown in Figure 1a. A human serum sample containing both PCT and CRP antigens was added to the sample pad and migrated from left to right. Two detection antibodies: a mouse anti-human CRP monoclonal antibody (CalBioreagents, Cat # M353) and a mouse anti-human PCT monoclonal antibody (MyBioSource, Cat # MBS310731, Clone # 42), were conjugated to EuNPs before being placed on the conjugate pad using the protocol described above.

All test and control antibody solutions were diluted in 1X PBS buffer to 0·5 mg/mL. They were then dispensed onto the nitrocellulose membrane with an Automated Lateral Flow Reagent Dispenser at a rate of 6·7 mL/min. Three parallel lines were printed to the membrane, each 3 mm apart: the first or the bottom line for CRP capture with a mouse anti-human CRP monoclonal antibody (CalBioreagents, Cat # M354), the second or the middle line for PCT capture with a mouse anti-human PCT monoclonal antibody (MyBioSource, Cat # MBS310737, Clone # 16B5), and the third or the top line with a goat polyclonal antibody anti-mouse to serve as the control antibody. Membranes were immediately placed in the 37 °C incubator for 1 h and later transferred to a desiccator. The EuNP-conjugated antibody solution was diluted 20 times to a concentration of 0.05% with 1X TBS buffer containing 1% BSA, 1% Tween 20, and 2% sucrose. For each test strip, 5 µL PCT conjugation antibody solution (0.05%) and 5 µL CRP conjugation antibody solution (0.05%) were applied. The test strips were cut into strips of 4 mm using a paper trimmer before being assembled in plastic cassettes.

In clinical testing with patient serum samples, testing was carried out with 25 µL of the specimen. For the 1 to 10 diluted sample, 2·5 µL of the specimen was mixed with 22·5 µL of 1X PBS solution. The reaction was initiated by pipetting specimen on the sample pad, followed by a buffer wash (Supplementary Fig. 1b). PCT and CRP molecules (if present) were captured on test lines by their respective capture antibodies beforehand immobilized onto the nitrocellulose membrane during the upward capillarity migration. Unbound and excess complexes were washed away with 85 µL of 1X TBS wash buffer containing 1% BSA, 1% Tween20, and 2% sucrose. After ∼20 min incubation in the dark, the fluorescence signal of the test strips was read on a custom-built UV fluorescence-based optical reader. Sample cases for various detection scenarios, from test malfunction at co-detection, were included in Supplementary Fig. 1a.

### Optical reader design

**Figure 2:**
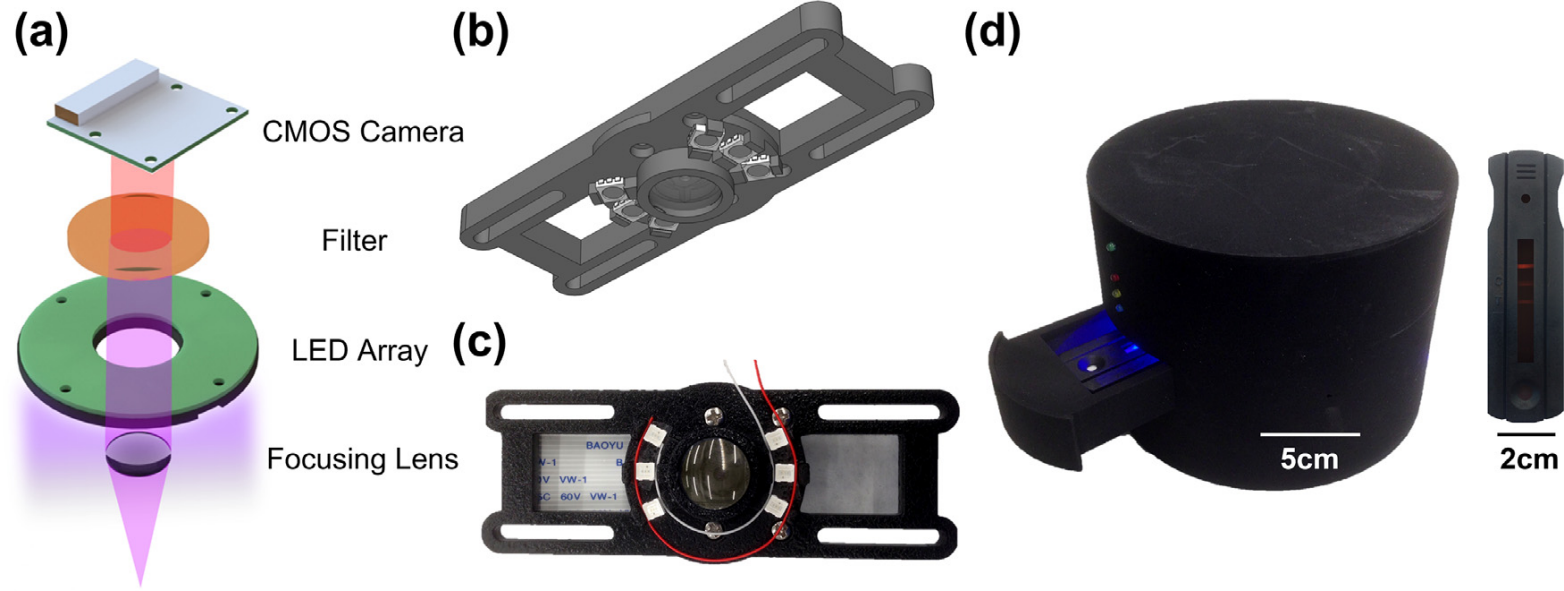
Inner assembly of the design of the UV fluorescence-based optical reader. (a) Exploded view of the optical reader. The fluorescence sensor excites the fluorescence signal with a 380 nm UV LED and captures the spectrum larger than 530 nm in wavelength with a long pass filter. The CMOS camera and the LED array are controlled by a Raspberry Pi inside the optical reader. The sensor holder and the Raspberry Pi are not shown in the figure for clarity. (b) CAD design for the UV fluorescence sensor coupled with other parts. (c) Assembly view of the UV fluorescence sensor coupled with other parts. (d) External view of the UV fluorescence-based optical reader with casing. The PCT/CRP duplex LFA assembled in a cassette is placed on the sliding tray. During the imaging process, the indicator light is turned on, and the tray is closed to remove the effect of external light. The picture of the LFA shows the UV fluorescence signal on the test strip for a co-detection case where the control line, PCT test line, and CRP test line all show up from top to bottom.

Figure 2 shows the inner assembly of the design of the UV fluorescence-based optical reader. Key components of the optical reader include a CMOS camera, a long pass filter, an LED array, and a focusing lens, as shown in Figure 2a. The fluorescence sensor excited the fluorescence signal with 380 nm UV and captured the spectrum above 530 nm in wavelength with the filter. The CMOS camera, the LED array, and the data transmission unit were controlled by a Raspberry Pi inside the optical reader. The data transmission was achieved via a Wi-Fi adapter to transfer raw data of the CMOS camera to a computer. Figure 2b and c show the CAD design and assembly view of the UV fluorescence sensor coupled with other parts. An external view of the UV fluorescence-based optical reader with casing is depicted in Figure 2d with scale bars to show the dimensions of the reader and the test strip cassettes. It also shows the UV fluorescence signal on the test strip for PCT/CRP co-detection. The custom-built optical reader has two distinct advantages. First, the easy-to-use reader is built off low-cost commercially available components at a total price of below $70, similar to that of the latex-bead-based reader that we previously reported.^41^ We envision such a reader system as a piece of affordable medical equipment requiring minimal training to help physicians make management decisions at clinics in low-resource settings. Second, the reader system stores unprocessed raw data of the CMOS camera and directly correlates biomarker concentrations with the raw data. This offers flexibility to tune the brightness levels to find the optimal value and apply the same brightness setting to all test cases to avoid background disparity by camera autocorrection. We have included test strip images with different background brightness after adjusting the brightness settings to extract images from the same unprocessed raw data in Supplementary Fig. 2. We notice that as the brightness level increases, the test line intensity also increases until it reaches saturation. An optimal brightness value needs to be determined and applied to all test cases to avoid background disparity without making the test line intensities too dim or saturated.

### Quantification of PCT and CRP antigens in spiked buffer test

Calibration curves for both PCT and CRP were generated with purified recombinant proteins in the spiked buffer test. In each case, proteins were serially diluted in 1X PBS buffer over various workable concentrations (0·5–100 ng/mL for PCT, and 0·05–10 mg/dL for CRP). The difference in the concentration units used herein was purposely chosen to account for the physiological differences in the baseline levels of these two biomarkers sera of healthy individuals in the absence of bacterial infection and other inflammatory insults. We ran the calibration experiments in triplicate and plotted the resulting data as the average of means ± standard deviation (SD) (mean ± SD, *n* = 3).

### Clinical sample validation

We focused on validation in the context of sepsis, where there is the greatest amount of data on the utility of these biomarkers. We acquired 25 remnant patient serum samples with known PCT and CRP concentrations from Weill Cornell Medicine (New York, NY, USA). These patients’ samples covered the whole physiological range for sepsis, including: local infection (0·05–0·5 ng/mL], sepsis (0·5–2 ng/mL], severe sepsis (2–10 ng/mL], and septic shock (>10 ng/mL). The concentrations of PCT and CRP were measured using FDA cleared methods: Roche Cobas e411 Elecsys PCT and Siemens ADVIA Chemistry XPT CRP assays.

### Image processing protocol

Test strip images were processed with ImageJ software and LabVIEW.^42^^,^^43^ RGB values of a selected region of interest (ROI) of the LF strip signal were generated with ImageJ, followed using a numerical integration process for the grayscale peak areas using Simpson's 3/8 rule in LabVIEW.^44^^,^^45^ The peak areas were related to the test line intensities. The same ROI was applied to different test strip images to ensure consistency. The effect of ROI selection on the quantification capability of the PCT/CRP duplex platform is shown in Supplementary Figure 3.

### Statistical analysis

Measured grayscale peak area values from LFA tests were computed and plotted as average mean ± SD. The concentration levels for PCT and CRP were predicted by comparing the peak area values with their respective calibration curves. A correlation analysis was conducted to compare the LFA-predicted concentrations with results from gold-standard methods (Roche Cobas e411 for PCT and Siemens ADVIA Chemistry XPT for CRP).

### Role of funding source

This study was supported by the National Institutes of Health Award 1R01EB021331, and the Atkinson Center for a Sustainable Future through the Academic Venture Fund. The funders did not have any role in study design, data collection, data analyses, interpretation, or writing of the report.

## Results

### PCT quantification in spiked buffer

**Figure 3:**
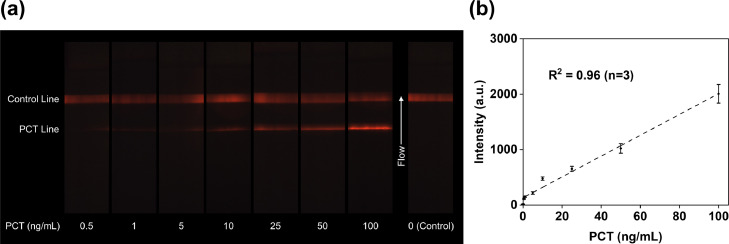
Test strip images and quantification results of PCT antigens spiked in running buffer. (a) Images of test strips with PCT concentrations from 0·5 ng/mL to 100 ng/mL. The PCT test line intensity increases under rising concentrations. The control line intensity shows variation due to the different amounts of excess labelled antibody conjugation captured in the control zone under different PCT concentrations. Since the final PCT/CRP duplex test contains two test lines, the control line only validates the strip's function. Only the test lines intensities are analyzed for further quantification. (b) Relationship between PCT test line intensities and PCT concentrations. The linear fitting generates a good correlation, with an R^2^ value of 0·96. Error bars represent standard deviations of the triplicate measurement means.

PCT antigens with concentrations ranging from 0·5 to 100 ng/mL in spiked buffer and a blank control sample were imaged with the UV fluorescence-based optical reader, as shown in Figure 3a. The limit of detection (LoD) of PCT analytically achieved in buffer was 0·5 ng/mL, a value close to the baseline PCT level in healthy individuals. Figure 3b presents the calibration curve in the buffer test: PCT test line intensities were correlated with PCT antigen concentrations in the spiked buffer. A linear fitting was established to fit the relationship between PCT test line intensities with concentrations to derive a function such that PCT intensity = f [PCT] where [PCT] is the PCT antigen concentration in spiked buffer, with an R^2^ value of 0·96, as shown in Figure 3b. In this concentration range, PCT test line intensity increased as the concentration rose. The PCT triplicate intra-assay variability in spiked buffer test ranged from 6·8% (0·5 ng/mL) to 11·0% (5 ng/mL), as shown in Supplementary Table S1. We also confirmed that the LFA strip did not bind to calcitonin antigens, proving the assay's specificity for binding to PCT antigens only.

### CRP quantification in spiked buffer

**Figure 4:**
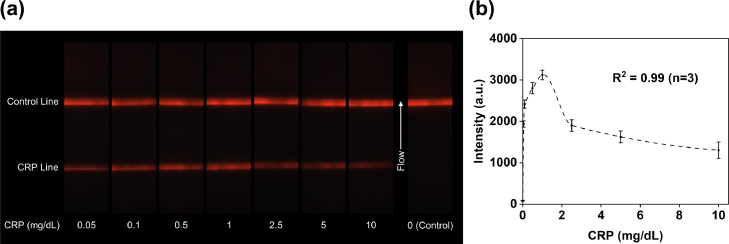
Test strip images and quantification results of CRP antigens spiked in running buffer. (a) Images of test strips with CRP concentrations from 0.05 mg/dL at 10 mg/dL. The CRP test line intensity changes under different concentrations. When CRP concentration is low, the signal intensity rises with concentration until it reaches the hook effect. Beyond this point, a further increase in the concentration decreases the signal intensity. (b) Correlation between CRP test line intensity and CRP concentrations. CRP signal reaches hook effect at 1 mg/dL. Error bars represent standard deviations of the triplicate measurement means.

CRP antigens with concentrations ranging from 0·05 to 10 mg/dL in spiked buffer and a blank control sample were imaged with the UV fluorescence-based optical reader (Figure 4a). The analytical LoD of CRP was estimated to be 0·05 mg/dL. The quantification result in the buffer test was presented in Figure 4b, where CRP test line intensities were correlated with CRP antigen concentrations in the spiked buffer. An Akima spline interpolation fitting was established to fit the relationship between CRP test line intensities with concentrations to derive a function such that CRP Intensity = f [CRP] where [CRP] is the CRP antigen concentration in spiked buffer, with an R^2^ value of 0·99, as shown in Figure 4b. According to this curve, CRP test line intensity increased with the rising concentration below 1 mg/dL. Above 1 mg/dL, CRP test line intensity decreased as the concentration further increased. This decrease in test line intensity at higher concentrations has been described as the high-dose hook effect caused by excess unlabeled analytes occupying binding sites at test lines.^46^ For a given CRP test line intensity, one can usually expect to get two concentration values from the calibration curve, leading to a dilemma in predicting the correct value. For instance, the CRP test line intensity value at 2·5 mg/dL was similar to 0·05 mg/dL. Various approaches have proved useful in solving the “quantification dilemma” brought by the hook effect, such as reaction kinetics,^47^ serial dilution,^48^^,^^49^ or adding a competitive test line.^50^ The CRP triplicate intra-assay variability in spiked buffer test ranged from 3·7% (1 mg/dL) to 15·1% (10 mg/dL), as shown in Supplementary Table S1.

### Method comparison using human serum samples

**Figure 5:**
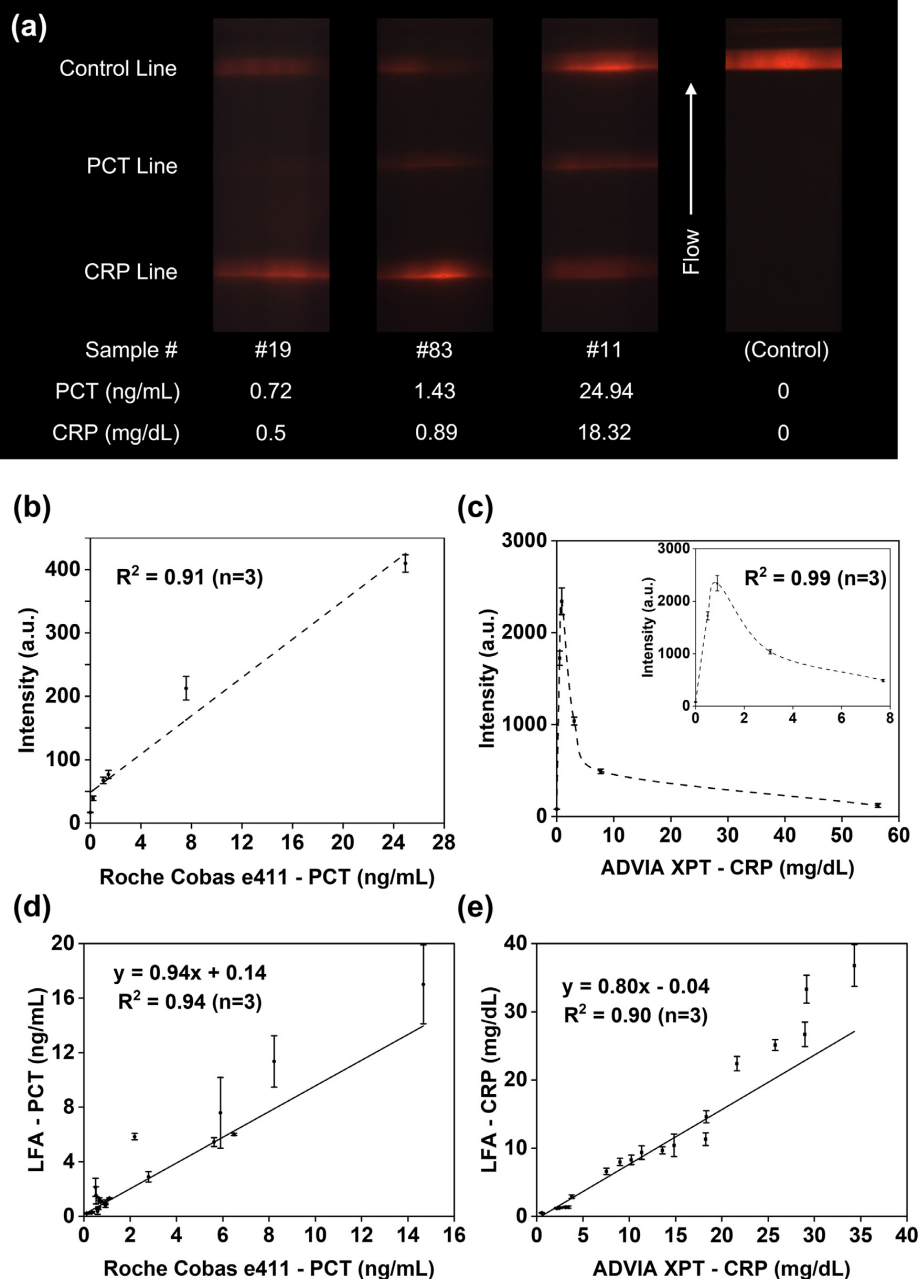
Method comparison for both PCT and CRP using human serum samples. (a) Three test cases of clinical samples and a negative control using a blank sample. PCT test line intensity increases with the rising concentration, whereas CRP test line intensity first increases as the concentration rises until it reaches the hook effect range. After a specific concentration, a further increase in CRP concentrations leads to decreased test line signal intensity. (b) Calibration curves for PCT antigens in human serum using the gold standard results of five clinical samples. (c) Calibration curves for CRP antigens in human serum using the gold standard results of five clinical samples. (d) Correlation plot of predicted serum PCT concentrations with that by the gold standard method, Roche 4e11. (e) Correlation plot of predicted serum CRP concentrations with that by the gold standard approach, ADVIA XPT. All error bars in (b)–(e) represent standard deviations of the triplicate measurement means.

To validate the accuracy of the duplex LFA, we tested 25 serum samples previously tested in a clinical setting with FDA cleared methods: Roche Cobas e411 Elecsys PCT and Siemens ADVIA Chemistry XPT CRP. The performers of the duplex LFA were not given access to the clinical assay results before running the tests. All samples were run in triplicate, and the test line intensities were later quantified from the duplex LFA strip images taken by the custom-built UV fluorescence-based optical reader. As shown in Figure 5a, PCT test line intensities increased as the concentration rose for the range of all clinical samples. CRP intensities, however, first rose with increasing concentrations, and the signal got dimmer at higher concentrations, similar to what we observed in the spiked buffer test in the previous section. Because of this, a given CRP test line intensity is often associated with two concentration values. To determine the correct concentration at high concentrations, our group has previously demonstrated a real-time reaction kinetics-based method to mitigate the hook effect by one test strip.^47^ To bypass monitoring the reaction kinetics for all strips, here we adopted a serial dilution as a simple confirmation step to verify the result. Examples to show the detailed protocol are included in Supplementary Fig. 4.

According to the clinical assay results, sample #4 had the PCT concentration at 199·5 ng/mL, while the PCT concentrations for the remaining 24 samples ranged from 0·22 to 24·94 ng/mL. We excluded sample #4 from the statistical analysis for the following reasons. First, the PCT concentration of sample #4 differed significantly from other observations. Second, the remaining 24 samples already had a balanced distribution covering the whole physiological range from local infections to septic shock. Third, we optimised our LFA components and validated its PCT quantification range up to 100 ng/mL. All the remaining 24 serum samples were from patients. Supplementary Table 2 summarizes the different stages of sepsis of these samples based on their PCT levels characterized by Roche Cobas e411 Elecsys PCT. Due to the differences in standard curves for different mediums such as running buffer and human serum,^51^ we randomly selected five serum samples with concentrations covering the whole detection range for both PCT and CRP from the remaining 24 samples to generate a standard curve for tests in human serum. We compared the clinical assay results for the five samples with PCT and CRP test line intensities calculated from LFA tests, and generated calibration curves in serum for PCT and CRP, respectively, as shown in Figure 5b and c. The two calibration curves were then used to predict the PCT and CRP values for the 19 remaining patient samples in human serum.

A correlation plot of serum PCT concentrations predicted from LFA results against corresponding FDA cleared method results by Roche Cobas 4e11 was presented in Figure 5d. Results showed a correlation of 0·97 (*P* < 0·01, linear regression), with an R^2^ value of 0·94 for a linear fitting. The PCT triplicate intra-assay variability in clinical validation test ranged from 1·3% (6·01 mg/dL) to 16·9% (17·01 mg/dL). Figure 5e shows the correlation plot of serum CRP concentrations predicted from LFA results against corresponding clinical method results by ADVIA XPT. Findings demonstrated a correlation of 0·95 (*P* < 0·01, linear regression), with an R^2^ value of 0·90 for a linear fitting. The CRP triplicate intra-assay variability in clinical validation test ranged from 3·1% (25·13 mg/dL) to 11·1% (10·43 mg/dL).

## Discussion

Bacterial infection and sepsis exert serious public health and economic burden worldwide. The ability to provide early and accurate diagnosis at the point of care via a rapid test that distinguishes bacterial from viral infection holds numerous benefits with great potential for improving patients’ outcomes, decreasing antibiotic misuse, and reducing healthcare costs. Current diagnostic platforms in clinical settings rely on expensive and lengthy protocols that are difficult to implement,^52^ reducing their potentials for applications in point of care settings.

In the efforts to address these shortcomings and limitations, innovative approaches have been attempted to detect bacterial infection indirectly via host biomarkers whose baseline levels are up-regulated during bacterial infection. These new approaches reduce the diagnostic time and provide physicians with accurate and actionable information. However, most of these approaches are rarely used at the point of care due to their complexity. PCT and CRP have gained high distinction in clinical settings among the biomarkers of interest due to their high diagnostic values for inferring bacterial infection and sepsis.^53^ Previous studies also showed that by developing a duplex test for simultaneous detection of both PCT and CRP antigens, we could increase the sensitivity and specificity for differentiating bacterial and viral infections at the point-of-care.

In this study, we developed a EuNP-based duplex lateral flow strip for rapid and simultaneous detection of PCT and CRP. We first built a UV fluorescence-based optical reader, which could pick up faint signals to increase the system's LoD. We first evaluated the assay performance in the spiked buffer to prove its quantification potential, followed by a clinical validation test with archived human serum samples.

In the spiked buffer test, we showed the LFA's quantification capability and ability to detect PCT concentrations at around 0·5 ng/mL, a value close to the baseline PCT level in healthy individuals.^54^ No crossreactivity was observed between PCT and calcitonin on the duplex LFA, suggesting that the assay was specific for PCT. Parallel to the PCT analysis, we covered a whole range of concentrations from 0·05 to 10 mg/dL in the CRP-spiked buffer test. Since the physiological concentration of CRP in healthy individuals is often < 1 mg/dL, the CRP concentration detected here strongly suggested the capability of the duplex to probe fluctuations levels of CRP during bacterial infections. To solve the difficulty in high-dose CRP quantification for sandwich-type LFAs, our group has previously demonstrated the feasibility of a real-time reaction kinetics-based method to mitigate the hook effect with a single test strip.^47^ This kinetic monitoring technique approach records the evolution of test and control line intensities over time. It uses the kinetics curve to overcome the hook effect and determine the right concentration. Therefore, we can use a single strip to accurately quantify CRP concentrations in the hook-effect range. But this kinetics-based approach requires keeping track of the test and control line development over 1000 s of reaction time, and conducting image analysis for all test strips during the reaction time was labor-intensive. Therefore, we adopted a simple serial dilution approach for verification purposes in the current study. The dilution factor was optimised as 1 to 10, so the total sample volume required to make a measurement was only 27·5 µL with two test strips, and the verification was easy to operate.

In the clinical method comparison with patient samples, we validated the LFA results for PCT and CRP concentrations against their corresponding method results by Roche Cobas 4e11 and ADVIA XPT, respectively. The Spearman's coefficients confirmed the diagnostic accuracy of the duplex for detecting the two biomarkers. Results showed a correlation of 0·97 (*P* < 0·01) for PCT, with an R^2^ value of 0·94 for a linear fitting, and a correlation of 0·95 (*P* < 0·01) for CRP, with an R^2^ value of 0·90 for a linear fitting.

The differences observed here in the concentrations of PCT and CRP measured using the FDA cleared methods versus those of the duplex could be attributed to many reasons, including macromolecular crowding and possibly hook effect.^47^ Additionally, intrinsic and inherent proprieties of the assay itself (e.g., affinity and avidity of antibodies, buffer compositions, etc.) could not be overlooked. Nonetheless, despite these minor incongruities, the duplex LFA qualitatively detected CRP and PCT in clinical specimens and showed good correspondence with FDA-cleared methods, indicating that the duplex LFA could potentially be used to monitor fluctuations of these two biomarkers. One possible application area is in the neonatal care unit (NICU), where bacterial infections of newborns are common. In fact, PCT levels alone have a 93% negative predictive value in excluding bacterial infection in neonates.^55^ Current laboratory techniques used in NICU rely on traditional microbiological cultures, polymerase chain reaction (PCR), and protein assays. Unfortunately, many of these techniques take hours or even days to return a definitive diagnostic result, which is not ideal in serious infectious situations when the babies’ lives are at stake. Additionally, since bacteria are dynamic in their growth characteristics, a diagnostic strategy that unnecessarily lengthens the turnover time will likely impede the physician's ability to diagnose the infection and administer treatment.

The primary innovation of this study is the rapid and simultaneous quantification for both PCT and CRP antigens over a large physiological range relevant to sepsis patients by a low-cost optical reader-based LFA system, making it suitable for applications in low-resource settings for viral and bacterial infection differentiation. One of the main difficulties for combining these two targets on the same LFA strip is that the physiological range of CRP is around one thousandfold higher than that of PCT. In addition to unique strip architecture and optimization, our custom-built UV fluorescence reader system has shown distinct advantages to enable accurate co-quantification with the following attributes. First, the reader applies a novel imaging mechanism to avoid background disparity by storing unprocessed raw data of the CMOS camera and applying the same brightness settings across multiple strips. Second, the reader system can also be easily adapted to visualize various conjugation materials. Our group has demonstrated that with minor modifications to the band-pass optical filters, the optical reader presented herein can be readily adapted for LFAs based on gold nanoparticle conjugation,^56^^,^^57^ latex beads,^41^ and fluorophores such as R-phycoerythrin (RPE), fluorescein (FITC), and phycoerythrin/Cyaine5 (PE/Cy5).^58^ Third, the optical reader optimised for colour detection and differentiation has the distinct advantages of picking up faint signals to increase the LoD for analytes. Finally, we calculated the cost for the optical reader and test strips. The reader was built with off-the-shelf components at a total price below $70, with over 60% of the cost from the Raspberry Pi and the focusing lens.^41^ Each duplex strip for PCT and CRP co-detection costs around $3·5 to manufacture (Supplementary Table 3), with EuNP accounting for more than 80% of the total cost. We anticipate that the test strip cost can be reduced dramatically by using alternative nanoparticles such as upper conversion nanoparticles, which are low-cost particles that have exhibited sensitivities similar to EuNPs.^59^ In addition to using alternative conjugation materials, optimization studies on the amount of antibody immobilized on the test lines (e.g., the amount of anti-PCT capture antibody) can also help reduce the strip cost further.

Meanwhile, we recognize that while the current study presents serial dilution as a simple approach for concentration verification, it inevitably increases the testing cost since two test strips will be required. We envision that by automatic image collection and subsequent data extraction, we can achieve real-time assay kinetics measurement more efficiently to use one single strip for mitigating the hook effect in sandwich-type LFAs. The optical reader-based duplex LFA's ability to quantify both PCT and CRP on the single test strip, combined with its low cost and rapid response, makes it suitable for use in clinical settings where timely and accurate diagnostics are warranted. Further optimization studies for the optical reader system can help reduce the cost for the system (e.g., by replacing the battery pack with chargeable modules can help save 8% of the system cost), and increase the imaging quality (e.g., by optimizing the image sensor module and imaging algorithm to pick up faint signals), thus improving the detection limits for target analytes.

A caveat of this study is its limited sample size and the difficulty in optimizing the assay parameters in serum due to the ubiquitous presence of these biomarkers in humans. Future studies with an adequate sample size will need to be performed to corroborate the findings of this study on a large scale. We also envision other bacterial infection-specific biomarkers, such as interleukin-6 (IL-6), can be incorporated into our multiplexed LFA platform to further increase the diagnostic accuracy for differentiating bacterial and viral infections. Admittedly, the low circulating level of IL-6 in healthy adults in the range of pg/mL could pose a detection challenge to LFA systems. However, during bacterial infections, the IL-6 concentration increases dramatically, and under severe conditions, it can even reach the μg/mL level,^60^ a range well within the detection limit to LFA systems. Several recent studies have demonstrated the efficacy of LFA-based systems in quantifying IL-6 in human serum during bacterial infections.^61^^,^^62^ To overcome the physical limitation of LFA test strips for multiplexing, our group has developed a colour encoding mechanism to combine multiple detection targets on the same test line. We first encode each target with a different colour and apply a colour separation algorithm to extract the colour types and intensities to inter the detection species and their concentrations.^41^^,^^44^ Meanwhile, clinical validation in the current study was conducted in human serum due to sample availability and the potential complications in directly testing whole blood with LFAs. Previous studies have demonstrated the interference by red blood cells and lysed fragments on LFA test line and control line signals when whole blood samples are directly applied to an LFA strip.^63^ To solve this problem, blood separation to extract serum from whole blood samples is needed. On the one hand, serum can be extracted by centrifugation after clotting, and low-cost blood centrifuges, such as a 20-cent, hand-powered blood centrifuge,^64^ have shown great potential for point-of-care applications. On the other hand, “on-strip” blood separation on LFAs through novel test strip design (e.g., the incorporation of a blood separation membrane/unit on an LFA strip)^65^^,^^66^ can also solve the complications of using whole blood samples on LFAs, and has presented another potential solution for clinics without access to centrifugation capabilities.

In summary, we developed a duplex lateral flow assay-based platform for differential diagnostics of bacterial infections via rapid and simultaneous quantification of PCT and CRP antigens in human serum over a large physiological range observed in sepsis patients, with a minimal sample volume requirement of 27·5 µL that can be readily obtained by a typical finger prick. We first optimised the assay by spiking antigens of known concentrations in the buffer, followed by a validation study with actual patient samples to prove a good correlation of our LFA assay's quantification results with the values from the FDA cleared methods. The quantification of the duplex LFA was achieved by a low-cost custom-built portable UV fluorescence reader, which blocked the influence of external lighting to ensure background uniformity. Finally, the fluorescence reader-based duplex LFA demonstrates several advantages, including portability, rapid response (∼20 min), low cost (∼$3·5), making it well suited for use in point-of-care clinical settings for differentiating bacterial and viral infections.

## Declaration of interests

The authors have no conflicts of interest to disclose related to this work. In the interest of full disclosure, David Erickson and Saurabh Mehta are founders and board members for a diagnostic start-up focused on measuring nutritional biomarkers at the point-of-care.

## Appendix Supplementary materials

Image, application 1
