# Supplementary material for: A diagnostic platform for rapid, simultaneous quantification of procalcitonin and C-reactive protein in human serum
Source: eBioMedicine. 2022 Feb 8;76:103867. doi: 10.1016/j.ebiom.2022.103867 (PMC8841998; doi:10.1016/j.ebiom.2022.103867)
Supplement: Supplementary file 1 [file mmc1.docx]

Supplementary materials

**A Diagnostic Platform for Rapid, Simultaneous Quantification of Procalcitonin and C-Reactive Protein in Human Serum**

Xiangkun Elvis Cao ^a,1^, Serge Y. Ongagna-Yhombi ^a,b^, Ruisheng Wang ^c^, Yue Ren ^a^, Balaji Srinivasan ^b^, Joshua A. Hayden ^d,2^, Zhen Zhao ^d^, David Erickson ^a,b,e,*^, and Saurabh Mehta ^b,e,*^

^a^ Sibley School of Mechanical and Aerospace Engineering, Cornell University, Ithaca, New York 14853, United States

^b^ Division of Nutritional Sciences, Cornell University, Ithaca, New York 14853, United States

^c^ Meinig School of Biomedical Engineering, Cornell University, Ithaca, New York 14853, United States

^d^ Department of Pathology and Laboratory Medicine, Weill Cornell Medicine, New York, New York, United States of America

^e^ Institute for Nutritional Sciences, Global Health, and Technology, Cornell University, Ithaca, New York, United States of America

^1^ Present address: Department of Chemical Engineering, Massachusetts Institute of Technology, Cambridge, Massachusetts, United States

^2^ Present address: Norton Healthcare, Louisville, Kentucky, United States

*Corresponding Authors

David Erickson (E-mail: [de54@cornell.edu](mailto:de54@cornell.edu))

Saurabh Mehta (E-mail: [smehta@cornell.edu](mailto:smehta@cornell.edu))

**Contents:**

**Supplementary Fig. 1.** Sample cases and protocol of running the PCT/CRP duplex lateral flow assay (LFA).

**Supplementary Fig. 2.** Test strip images with tunable background brightness.

**Supplementary Fig. 3.** The effect of ROI selection on the quantification of the PCT/CRP duplex LFA test strips.

**Supplementary Fig. 4.** Serial dilution as an affirmative step to determine CRP concentrations.

**Supplementary Table S1.** Intra-assay variability for PCT and CRP quantification from triplicate tests in spiked buffer.

**Supplementary Table S2.** Different stages of sepsis of 24 clinical samples based on their PCT concentrations characterized by Roche Cobas e411 Elecsys PCT.

**Supplementary Table S3.** Cost breakdown of PCT/CRP duplex LFA test strip.


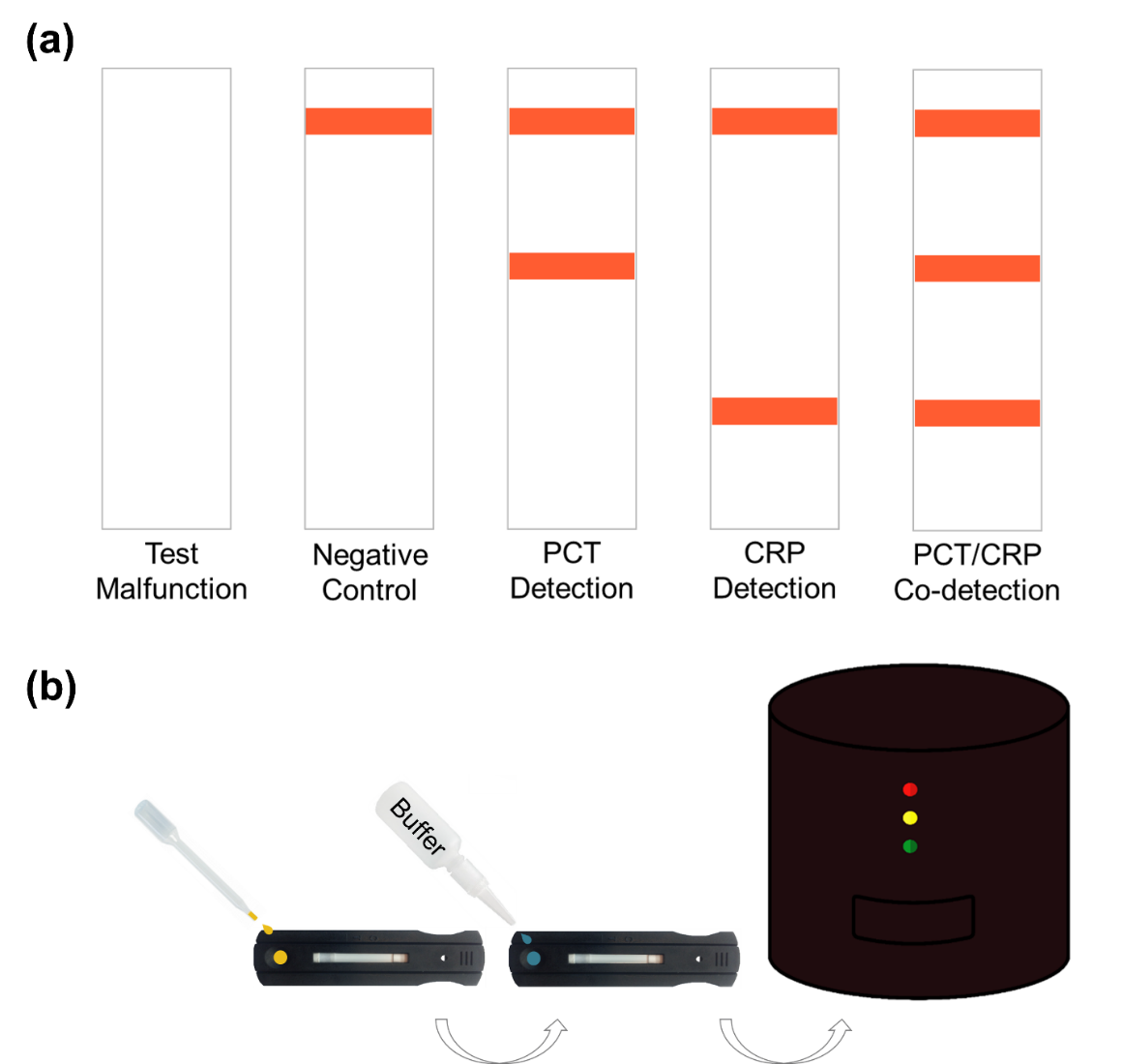


**Supplementary Fig. 1**

Sample cases and protocol of running the PCT/CRP duplex lateral flow assay (LFA). **(a)** Sample cases for various diagnostic scenarios. **(b)** Protocol of the PCT/CRP LFA testing. A sample containing the analytes is added to the sample pad. After putting the running buffer, the test strip is inserted into the customer-built UV fluorescence-based optical reader. The reader is irradiated by ultraviolet (UV) light source to visualize the europium-based LFA.


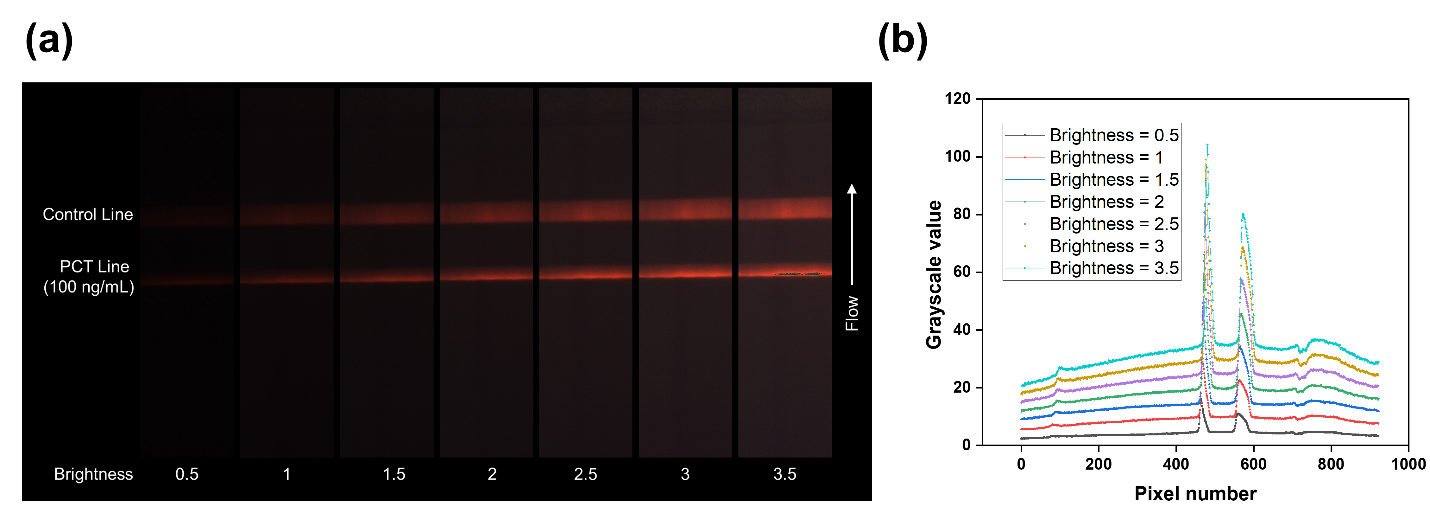


**Supplementary Fig. 2**

Test strip images with tunable background brightness. **(a)** These images were extracted after changing the brightness settings to the same unprocessed raw data (with the extension name of .data). This file is linked to the same test case (PCT: 100 ng/mL). As the brightness level increases, the PCT test line signal increases until saturation (Brightness = 3·5). As the brightness level decreases, the PCT test line signal decreases and gradually diminishes. A vital advantage of the UV fluorescence-based optical reader system is the ability to store unprocessed raw data of the CMOS camera. This offers flexibility to tune the brightness levels to find the optimal value and apply the same brightness setting to all test cases to avoid background disparity. **(b)** Comparison of average grayscale values generated from the seven different brightness settings. As brightness level increases, the peak area for PCT signals also increases, and PCT test line signal intensity (i.e., peak area) becomes stronger until saturation (Brightness = 3·5). At a low brightness level of 0·5, the PCT test line signal becomes very faint. Even after background subtraction, the grayscale peak area varies under different brightnesses. The optimal brightness should be set when the strongest test line signal is not saturated (Brightness = 3·0). Such a setting can help pick up faint signals and increase quantification accuracy. We also confirmed that none CRP signals get saturated at the brightness of 3·0. Therefore, the optimal brightness level is pre-set as 3·0 for all future processing.


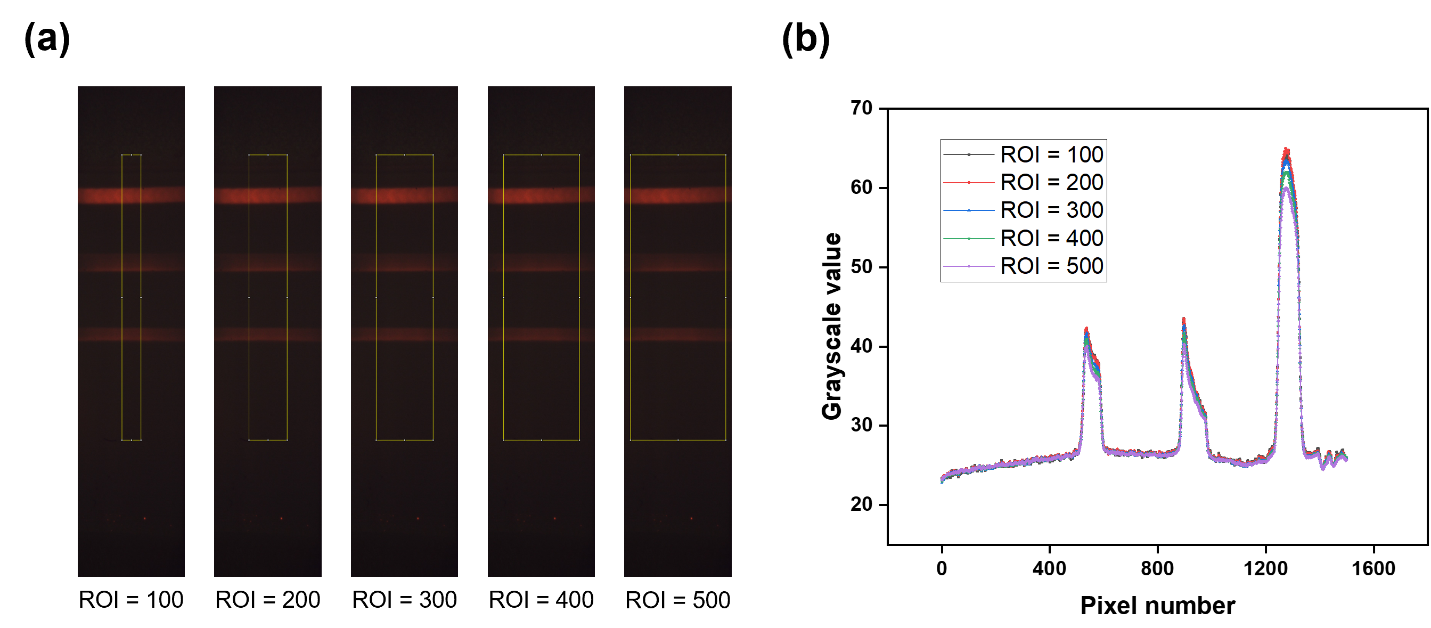


**Supplementary Fig. 3**

The effect of ROI selection on the quantification of the PCT/CRP duplex LFA test strips. **(a)** Comparison of five ROIs applied to the same image. **(b)** Comparison of average grayscale values generated from the five ROIs.

**
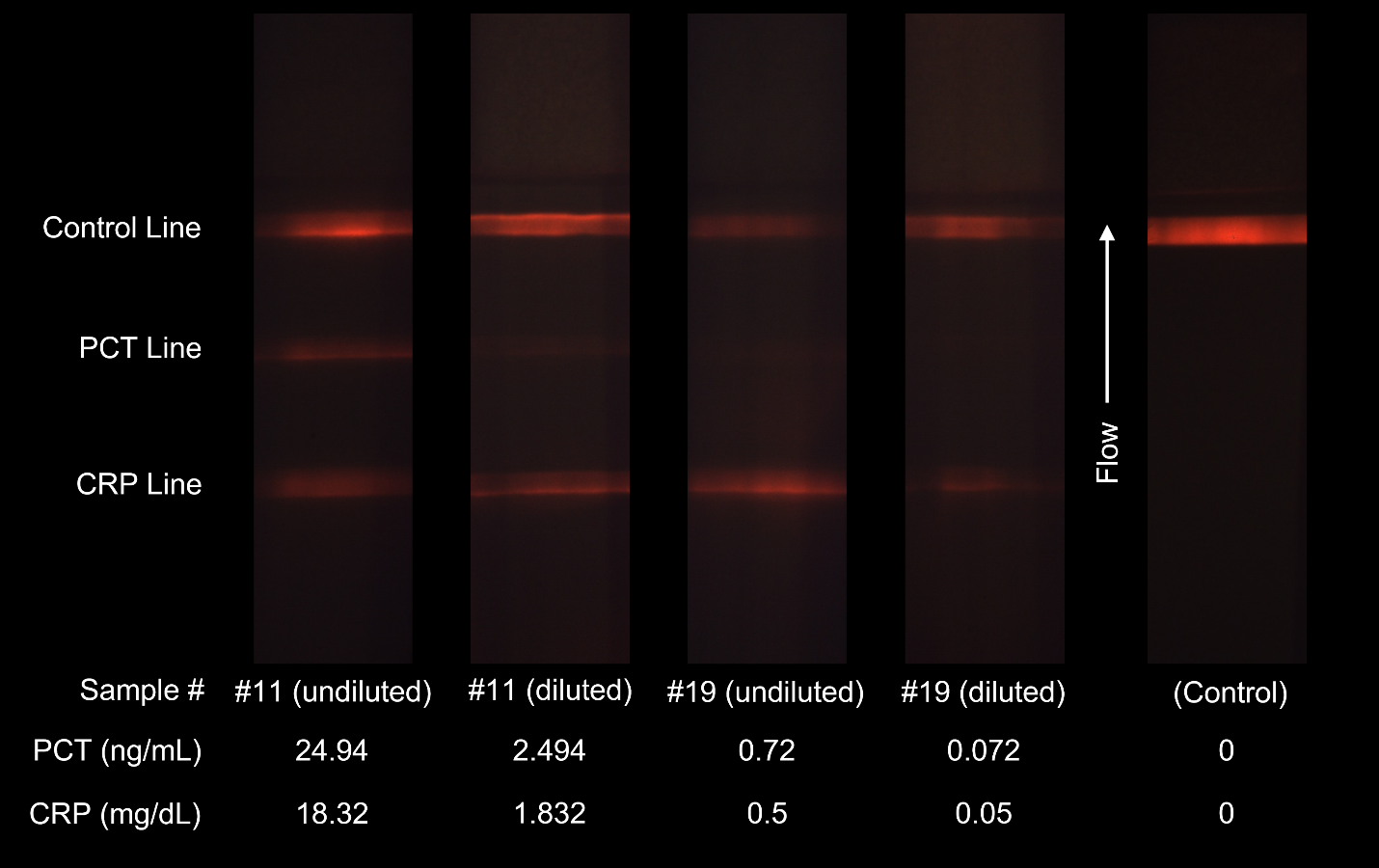
**

**Supplementary Fig. 4**

Serial dilution using two test strips as an affirmative step to determine CRP concentrations. The change in brightness between diluted and undiluted samples can be seen from the two sample cases. For our operating range, PCT intensity always goes down with dilution. In contrast, CRP intensity might rise or fall, depending on which side of the hook effect the sample concentration falls into. For Sample # 11, the 1/10 diluted sample has a dimmer PCT test line intensity but a stronger CRP signal when compared to that of the undiluted sample. For Sample # 19, both PCT and CRP intensities are dimmer for 1/10 diluted samples. We first use the undiluted sample to generate two concentrations values for a given CRP test line intensity. Then we run a serial dilution test and generate another image of the test strip. The CRP test line intensity of the diluted sample is then extracted, which also leads to two values. By comparing the concentrations values between diluted and undiluted samples, we then determined the correct CRP concentration of the sample.

**Supplementary Table 1.** Intra-assay variability for PCT and CRP quantification from triplicate tests in spiked buffer.

| **PCT (ng/mL)** | **Average grayscale intensity (a.u.)** | **Intra-assay variability** |
| --- | --- | --- |
| 0.5 | 110.99 | 6.8% |
| 1 | 150.37 | 7.3% |
| 5 | 218.39 | 11.0% |
| 10 | 477.02 | 7.3% |
| 25 | 635.16 | 6.9% |
| 50 | 1022.79 | 8.4% |
| 100 | 2008.37 | 8.2% |

| **CRP (mg/dL)** | **Average grayscale intensity (a.u.)** | **Intra-assay variability** |
| --- | --- | --- |
| 0.05 | 1933.90 | 3.9% |
| 0.1 | 2417.04 | 4.1% |
| 0.5 | 2799.21 | 4.8% |
| 1 | 3122.14 | 3.7% |
| 2.5 | 1899.91 | 7.4% |
| 5 | 1624.78 | 8.8% |
| 10 | 1302.73 | 15.1% |

**Supplementary Table 2.** Different stages of sepsis of 24 clinical samples based on their PCT concentrations characterized by Roche Cobas e411 Elecsys PCT.

| **Clinical stage** | **Concentration range (ng/mL)** | **# Number of samples** |
| --- | --- | --- |
| Healthy | < 0.05 | 0 |
| Local infections | (0.05-0.5] | 5 |
| Sepsis | (0.5-2] | 10 |
| Severe sepsis | (2-10] | 7 |
| Septic shock | > 10 | 2 |

**Supplementary Table 3.** Cost breakdown of PCT/CRP duplex LFA test strip.

| **Item** | **Cost** | **# Strips** | **Cost per strip** |
| --- | --- | --- | --- |
| Sample pad | $27 | 7500 | $0.004 |
| Conjugate pad | $32 | 7500 | $0.004 |
| Absorbent pad | $27 | 7500 | $0.004 |
| Nitrocellulose membrane | $344 | 7500 | $0.046 |
| Anti-mouse control Ab (1 mg) | $127 | 1800 | $0·071 |
| Anti-PCT capture Ab (1 mg) | $680 | 1800 | $0.378 |
| Anti-CRP capture Ab (1 mg) | $100 | 1800 | $0.056 |
| Anti-PCT detection Ab – Cnj (1 mg) | $680 | 40000 | $0.017 |
| Anti-CRP detection Ab – Cnj (1 mg) | $100 | 40000 | $0.003 |
| Eu nanoparticles (4 reaction kit) | $953 | 320 | $2.978 |

**Total: $3.561**
